# Urban-rural disparities in smoking behaviour in Germany

**DOI:** 10.1186/1471-2458-6-146

**Published:** 2006-06-06

**Authors:** Henry Völzke, Hanne Neuhauser, Susanne Moebus, Jens Baumert, Klaus Berger, Andreas Stang, Ute Ellert, André Werner, Angela Döring

**Affiliations:** 1 Institute of Epidemiology and Social Medicine, University of Greifswald, Germany; 2 Robert Koch-Institute Berlin, Germany; 3 Institute for Medical Informatics, University of Essen, Germany; 4 Institute of Epidemiology, GSF National Research Centre for Environment and Health Neuherberg, Germany; 5 Institute of Epidemiology and Social Medicine, University of Muenster, Germany; 6 Institute of Medical Epidemiology, Biometry and Informatics, Martin Luther University of Halle-Wittenberg, Germany

## Abstract

**Background:**

It is currently not clear whether individuals living in metropolitan areas differ from individuals living in rural and urban areas with respect to smoking behaviours. Therefore, we sought to explore the relation between residential area and smoking behaviours in Germany.

**Methods:**

We used a nationwide German census representative for the general population of Germany. A number of 181,324 subjects aged 10 years or older were included. Information on the average daily usage of cigarettes that have or had been smoked formerly or currently was available in subjects who have ever smoked. A daily consumption of more than 20 cigarettes was considered heavy smoking. Logistic regression analyses were performed sex-stratified and adjusted for relevant confounders.

**Results:**

Analyses revealed inhabitants of metropolitan areas to be more likely current smokers than inhabitants of rural areas (odds ratio 1.56, 95%-confidence interval 1.51; 1.62). Among current and former smokers those who lived in urban communities had also increased odds for being heavy smokers than those who lived in rural communities.

**Conclusion:**

We conclude that living in an urban and particularly living in a metropolitan area is a determinant of both smoking and severity of current smoking. Tobacco control programs should recognize the difference in living conditions between rural and urban areas.

## Background

Life in urban areas might be more stressful than life in rural areas. Populations that experience higher levels of stressful events have higher proportions of current smokers who also smoke more heavily than populations with respective lower levels [1]. This health behaviour model of stress in which populations under stress engage in behaviour which is highly detrimental to health has repeatedly been demonstrated in the context of low income and social status which may lead to an increased risk of smoking [2-5]. Communities can produce stress in individuals but can also provide the coping resources that help modify these stressors. Therefore, it is *a priori *not clear whether a higher or even a lower prevalence of smoking can be assumed in individuals living in urban areas compared to those living in rural areas. In particular the association between living in metropolitan regions and smoking has yet received sufficient attention. Knowledge on such differences would be of great relevance for smoking prevention programs, which have been demonstrated to be highly effective [6].

Previous studies [7-14] on this topic were predominantly performed in North America [7-13] and yielded conflicting results. Some studies [7,8,14] revealed subjects from urban areas to be more commonly current smokers than subjects from rural areas. These findings, however, have not always been confirmed by others [12] and further studies [9-11,13] revealed even the opposite, namely that individuals from rural areas to be at a higher risk of being smokers than individuals living in urban areas. Limitations of previous studies include inconsistent definitions of urban and rural areas, relatively small study populations, and reduced representativeness through investigations of selected populations thereby compromising the comparability as well as generalizability of those findings.

Moreover, there is a considerable lack of information regarding smoking habits in metropolitan compared to rural and urban regions. The mortality attributable to tobacco smoking in German Federal States is particularly high in states with a high proportion of metropolitan areas such as Berlin and Hamburg [15]. Likewise, a higher incidence of lung cancer has been demonstrated in metropolitan compared to urban or rural German regions [16]. Although several factors including general air pollution might explain these findings, it might also indicate a higher proportion of smokers in metropolitan regions.

The present study was designed to investigate the relation between residential area and smoking behaviours in Germany using data of the nationwide German Microcensus.

## Methods

### Study population

For this study, we used data of the Microcensus 1999 that represents a household sample representative for residents in Germany across the whole age range [17]. The sample was selected using population registries in which every resident's address, age and sex is included by law. For the survey, 1% of the households in Germany were randomly sampled. Selected subjects were legally obligated to participate in the Microcensus. Data were collected in April 1999. A number of 506,897 individuals took part in the survey, corresponding to a response proportion of 97%. A health-related questionnaire was applied to 50% of the sample which was also randomly selected. Questions on smoking behaviour were addressed to 210,268 participants aged 10 years or older. No information on current smoking behaviour was available from 28,944 individuals leaving a study population of 181,324 subjects (item-specific response proportion 86.2%) who were available for analyses. Informed written consent was obtained from all participants. The study was approved by the Ethics Committee and public data protection agencies.

### Measurements

Data on smoking behaviours were collected by personal interviews. With regard to current and former smoking, participants were questioned for smoking with respect to all tobacco products. Information on the average daily usage of cigarettes that have or had been smoked formerly or currently was available in subjects who have ever smoked. A daily consumption of more than 20 cigarettes was considered heavy smoking. We further selected potential confounders for the association between residential area and smoking behaviours. Education was categorized into three levels (<10 years, 10 years, >10 years) according to the German three-level schooling system. The current marital status comprised four categories (never married, married, divorced, widowed). The net income per capita was divided into four categories (<1000 DM, 1000 – <2500 DM, 2500 – <4000 DM, ≥ 4000 DM, 100 DM = 51.13 Euro). Subjects had their residency in East Germany if they lived in a region that belonged to the former German Democratic Republic including Berlin. This variable was considered as potential confounder because there were considerable changes in smoking behaviour among East Germans following the re-unification of Germany [14,15] and at the same time descriptive statistics revealed an association between residency in East Germany and urbanicity in our data:

### Statistical analyses

The study population was divided into subjects living in rural (<20,000 inhabitants), urban areas whereby the latter category was divided in two subcategories comprising small cities (20,000 – 500,000 inhabitants) and metropolitan areas (>500,000 inhabitants). The Microcensus originally covered five categories for defining community sizes: <5000, 5000–<20,000, 20,000 – <100,000, 100,000 – 500,000 and >500,000 inhabitants). The choice of the classification used in our study was based on the complete availability of the three aforementioned levels for all of the 16 German Federal States. Comparisons between rural and urban communities were made using logistic regression analysis (nominal data) or analysis of variance (ANOVA) (interval data) as appropriate. Multivariable logistic regression analyses were performed adjusted for age (decades), sex, marital status (4 categories), school education (3 categories), income (4 categories) and residency in East Germany. Odds ratios (OR) were calculated, values are given with 95%-confidence intervals (CI). A value of p < 0.05 was considered statistically significant. All statistical analyses were performed with SPSS software, version 11.5 (SPSS GmbH Software, Munich, Germany).

## Results

Selected characteristics of subjects who completed the health-related questionnaire of the Microcensus are given in table 1. Subjects living in urban communities were less often of male, better educated and more often single or widowed compared to subjects living in rural communities. Furthermore, there was a lower proportion of subjects with the lowest income and a higher proportion of East Germans among individuals from metropolitan areas compared to individuals from rural areas.

With regard to smoking habits, there was a higher proportion of current smokers and a lower proportion of never-smokers in urban communities compared to rural ones (Table 2). Albeit in part statistically significant, the starting age and the type of tobacco products used differed only marginally with respect to residential area. There was a higher proportion of subjects who smoked more than 20 cigarettes daily in urban compared to rural communities. All the differences between urban and rural communities were most pronounced for the comparison between metropolitan and rural areas (Table 2).

**Figure 1a-c:**
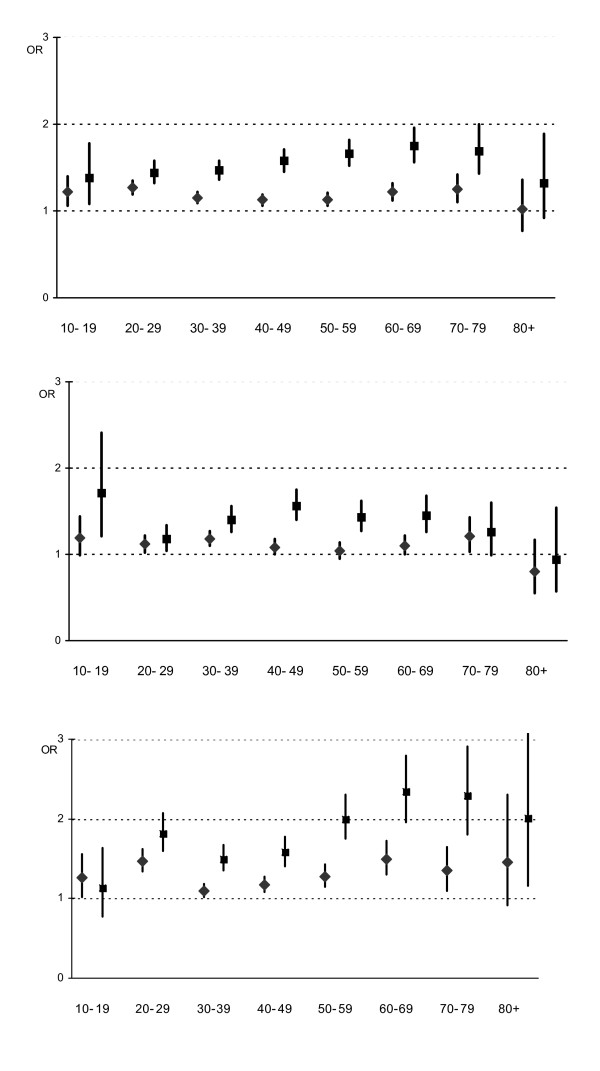
Odds of current smoking among individuals living in small cities (◆) and metropolitan (■) areas compared to inhabitants of rural areas. Results are given for the whole study population (Figure 1a) as well as the male (Figure 1b) and the female (Figure 1c) subpopulations. Data are odds ratio and 95% confidence intervals. OR estimates were controlled for marital status, school education, income and residency in East Germany.

The association between urbanicity and current smoking remained after adjustment for potential confounders (Table 3). Age-stratified analyses yielded that inhabitants of metropolitan areas aged 40 – 79 years were particularly more likely to be smoker compared to individuals living in rural areas (Figure 1a). Other factors significantly related to current smoking in the total population were male sex, younger age (<50 years), marital status other than married, low school education, and a monthly income between 1000 and 4000 DM (Table 3). High school education was inversely associated with current smoking, whereas residency in East Germany was not related to current smoking. Sex-stratified analyses revealed a more pronounced relation between residential area and smoking in the female subpopulation (Table 3, Figure 1b and 1c). Among women, the differences between rural and metropolitan communities with respect to current smoking were present over all ages but particularly evident for subjects aged 50 years or older (Figure 1c). In men, a higher monthly income was inversely associated with current smoking, but there was a direct association in women (Table 3). Modifications of the logistic regression model (usage of age as continuous variable, alternative categorizations of income, usage of the specific state instead of East or West Germany) did not considerably alter the estimates for the association of interest.

Logistic regression analyses that were performed with the same set of confounders using ever-smoking as the dependent variable also revealed an association between the size of the population and the endpoint. Compared to subjects living in rural communities, both subjects with residency in small cities and metropolitan communities exhibited higher odds for being ever-smokers (OR 1.18, 95%-CI 1.15; 1.21 and OR 1.53, 95%-CI 1.48; 1.59, respectively).

Among current and former smokers those who lived in urban communities also had increased odds for being heavy smokers in multivariable analyses. Compared to inhabitants of rural communities the OR for heavy smoking in individuals from small cities and metropolitan communities were 1.18 (95%-CI 1.16; 1.21) and 1.56 (95%-CI 1.51; 1.62), respectively. Compared to subjects from rural areas, women from metropolitan areas had higher odds for ever smoking (OR 1.79, 95%-CI 1.70; 1.87) than men (OR 1.36, 95%-CI 1.30; 1.43).

## Discussion

In the present study we applied data of the Microcensus 1999 to analyze the associations between residential area and smoking. While there was a relation between urbanicity and smoking in general, residency in metropolitan areas with more than 500,000 inhabitants was particularly related to higher odds of smoking compared to residency in rural areas. Furthermore, subjects living in urban areas were more often heavy smokers compared to those living in rural areas. Again, this relation was particularly evident for the comparison between rural and metropolitan areas. All investigated relations between urbanicity and smoking behaviours were present in both sexes but stronger among women than men.

Stress might be one explanation for urban-rural differences. Particularly in metropolitan areas this stress may be related to work as well as social factors. Work-related conditions may include longer commuting to work, a greater fear of becoming unemployed and a higher pressure to perform in larger companies. Social factors may include less social relations and a higher effort to find and to foster social contacts. These factors may affect women more than men [18] thereby explaining sex-related differences found in our study. This is in general concordance with results from two other studies [8,14]. The first [8] demonstrated that 57% of urban women were current smokers compared to 43% or rural women, whereas in men no such differences were found. The latter study [14] revealed that women living in cities with more than 500,000 inhabitants smoke by a factor of 50% more commonly than women from other communities [14].

Stress, however, cannot be the only explanation for the associations found in our study. Thus, suicide rates are higher in rural than in urban regions, particularly among younger individuals [19]. Internal migration might further explain the relation between urbanicity and smoking. Speculatively, more smokers than non-smokers might move from rural to urban communities than vice versa. Even a more general explanation is possible: individuals who move from rural to urban communities might be more susceptible for risk behaviours than individuals who move from urban to rural communities or individuals who do not move at all. Moreover, the general susceptibility for risk behaviours might represent the underlying cause for such migration activities. Unfortunately, data on migration were not available in our study. Therefore, we were not able to analyse the highly interesting change of exposure over time in the present context.

A specific problem in Germany is the relative absence of effective activities in smoking prevention. In contrast to many other countries there are few restrictions regarding smoking and cigarette advertisement as well as availability of cigarette machines in public places. Since inhabitants of larger German cities are more exposed to these circumstances, the findings described in the present paper appear to be plausible and may be representative for countries with little anti-smoking activities. The specific political environment as well as stress and migration, however, might not fully explain the relations reported herein. Therefore, studies are needed to further explore mechanisms underlying the association between urbanicity and smoking and to investigate the change of this association under an environment of improving tobacco control.

The present results are in concordance with results of other studies [7,14] that also found higher prevalence of smoking in metropolitan compared to rural regions. Conflicting results [9-13] may have resulted from methodological reasons and cultural disparities. All of these studies [9-13] were conducted in Northern America where anti-smoking activities are much better established than in Germany [20]. The awareness of smoking-related health hazards might be higher among inhabitants of urban compared to rural communities under such specific political environment. Furthermore, one of the former studies [12] investigated selected populations such as Native Americans. Cultural and social aspects might hence further explain the discrepancies between this study [12] and ours. In addition, all other studies [9-13] recruited only adolescents or students whereas our analyses covered the population aged 10 years and older.

There are some limitations of the present study. First, the comparability of studies on the relation between urbanicity and smoking including is limited since no standardized and generally accepted definitions of rural, urban and metropolitan areas are available. Thus, the definition of rural populations varied from ≤ 5000 [14] to <50,000 [10] inhabitants per community and the definition of metropolitan populations from >50,000 [11] to >500,000 [14] inhabitants per community. Other studies [12] used the population density to classify urbanicity. The choice of the classification used in our study was based on complete availability of data in the three levels <20,000, 20,000 – 500,000 and >500,000 inhabitants for all 16 German Federal States. Second, **w**ith the present data we were unable to analyze potential differences between communities within the metropolitan areas. In urban areas diversity is found within the same community, whereas diversity is more pronounced across rural communities [11]. Since such diversities may be important for the design of tobacco control programs [5], further analyses of regionally conducted studies should address this issue. Third, this study shares with others the limitations inherent to cross-sectional data. Because of a lack of time sequence, the relations reported here, while robust, should not be interpreted as causal.

Some strengths of the present study merit consideration. These strengths include the large size of the study population used in the analysis. In particular the high precision of risk estimates reflects the latter point. Moreover, due to the very high response proportion in the Microcensus 1999, the present data are characterized by a high grade of representativeness.

## Conclusion

We conclude that living in an urban and particularly living in a metropolitan area is a determinant of both smoking and severity of current smoking. Given the high prevalence of smoking in Germany, general efforts should be made to intensify anti-smoking policy. Tobacco control programs should recognize the difference in living conditions between rural and urban areas.

## Abbreviations

OR Odds ratio,

CI Confidence interval

## Competing interests

The author(s) declare that they have no competing interests.

## Authors' contributions

HV has made substantial contributions to the conception of the analyses, performed the analyses, drafted the manuscript and has given final approval of the version to be published. HN has made substantial contributions to the interpretation of data, revised the manuscript for important intellectual content and has given final approval of the version to be published. SM has made substantial contributions to the interpretation of data, revised the manuscript for important intellectual content and has given final approval of the version to be published. JB has made substantial contributions to the interpretation of data, revised the manuscript for important intellectual content and has given final approval of the version to be published. KB has made substantial contributions to the interpretation of data, revised the manuscript for important intellectual content and has given final approval of the version to be published. AS has made substantial contributions to the interpretation of data, revised the manuscript for important intellectual content and has given final approval of the version to be published. UE has made substantial contributions to the interpretation of data, revised the manuscript for important intellectual content and has given final approval of the version to be published. AW has made substantial contributions to the interpretation of data, revised the manuscript for important intellectual content and has given final approval of the version to be published. AD has made substantial contributions to the analysis and interpretation of data, revised the manuscript for important intellectual content and has given final approval of the version to be published.

## Pre-publication history

The pre-publication history for this paper can be accessed here:



## Figures and Tables

**Table 1:** Selected characteristics of subjects aged 10 years or older living in communities with different numbers of inhabitants

	Rural communities <20,000 inhabitants n = 82,462			Urban communities 20,000 – 500.000 inhabitants n = 74,385			Metropolitan communities >500.000 inhabitants n = 24,477		
	Total	Men	Women	Total	Men	Women	Total	Men	Women
Sex (male); %	48.6	-	-	47.2*	-	-	46.7*	-	-
Age; years	45 ± 20	43 ± 19	46 ± 21	46 ± 20*	44 ± 19*	47 ± 21*	46 ± 20*	44 ± 19*	47 ± 21*
School education									
<10 years	57.4	56.6	58.2	52.0*	50.4*	53.4*	45.1*	43.6*	46.4*
10 years	28.3	26.6	30.0	26.4*	24.2*	28.2*	26.7*	24.1*	28.9*
>10 years	14.2	16.8	11.8	21.6*	25.3*	18.4*	28.3*	32.3*	24.7*
Marital status									
single	29.8	34.3	25.5	31.3*	35.2*	27.8*	34.1*	37.8*	30.8*
married	57.4	59.1	55.9	53.7*	56.8*	51.0*	49.4*	53.0*	46.3*
divorced	8.8	3.1	14.1	9.1*	3.2	14.4	9.0	3.1	14.1
widowed	4.0	3.5	4.5	5.9*	4.8*	6.9*	7.5*	6.0*	18.9*
Monthly income									
<1000 DM	35.4	20.8	49.0	32.7*	20.2	43.9*	27.9*	18.6*	36.0*
1000 DM – <2500 DM	39.5	38.2	40.7	39.2	35.7*	42.3*	38.8	34.2*	42.9*
2500 DM – <4000 DM	17.0	26.5	8.1	19.0*	28.2*	10.8*	22.3*	29.4*	16.2*
≥4000 DM	8.1	14.5	2.2	9.1*	15.9*	3.0*	10.9*	17.8*	4.9*
Residency in East Germany	24.2	23.8	24.5	20.2*	19.9*	20.5*	30.6*	30.7*	30.6*

German Microcensus 1999. Data are percentage and mean ± standard deviation.

100 DM = 51.13 Euro

* p < 0.05, logistic regression and ANOVA; references: total, male and female population living in rural communities

**Table 2:** Smoking behaviour of subjects aged 10 years or older living in communities with different numbers of inhabitants

	<20,000 inhabitants n = 82,462			20,000 – 500.000 inhabitants n = 74,385			>500.000 inhabitants n = 24,477		
	Total	Men	Women	Total	Men	Women	Total	Men	Women
Smoking status									
Current regular smoker	21.7	27.8	16.0	23.6*	29.0*	18.7*	28.0*	33.0*	23.6*
Current occasional smoker	3.2	3.5	3.0	3.6*	3.9*	3.3*	3.5*	3.7	3.4*
Former smoker	16.6	22.8	10.9	17.4*	23.1	12.3*	18.8*	23.3	14.9*
Never-smoker	58.4	46.0	70.1	55.4*	44.0*	65.7*	49.7*	40.1*	58.1*
Starting age	18.3 ± 5.1	17.9 ± 4.5	18.9 ± 6.0	18.6 ± 5.6*	18.1 ± 4.9*	19.4 ± 6.5*	18.6 ± 5.7*	17.9 ± 4.8	19.4 ± 6.6*
Type of tobacco products usually smoked †									
cigarettes	96.3	94.5	99.3	96.2	94.2	99.0*	96.5*	94.5	99.0*
cigars, small cigars	2.1	3.0	0.6	2.3	3.3	0.8*	2.1	3.2	0.8
pipes	1.6	2.4	0.2	1.6	2.5	0.2	1.3	2.3	0.2
Number of daily smoked cigarettes*									
<5	17.1	12.8	24.1	16.6	12.1*	22.7*	14.8*	10.9*	19.4*
5 – 20	67.3	67.9	66.3	66.1*	66.1*	66.1	63.8*	62.3*	65.7
21 – 40	12.3	15.4	7.2	13.7*	17.3*	8.7*	17.1*	21.6*	11.8*
>40	1.6	2.2	0.6	2.1*	2.9*	1.0*	2.6*	3.7*	1.3*

German Microcensus 1999. Data are percentage and mean ± standard deviation.

* p < 0.05, logistic regression and ANOVA, references: total, male and female population living in rural communities

† Current and former smokers

**Table 3:** Determinants of current smoking

	Total	Men	Women
Sex (ref. female)	1.92 (1.86; 1.97)	-	-
Age (ref. 10 – <20 years)			
20 – <30 years	1.13 (1.05; 1.22)	1.32 (1.19; 1.46)	1.05 (0.94; 1.18)
30 – <40 years	1.32 (1.22; 1.42)	1.58 (1.42; 1.76)	1.24 (1.10; 1.39)
40 – <50 years	1.13 (1.05; 1.23)	1.42 (1.27; 1.59)	1.00 (0.88; 1.13)
50 – <60 years	0.60 (0.55; 0.65)	0.82 (0.73; 0.91)	0.47 (0.41; 0.53)
60 – <70 years	0.31 (0.28; 0.33)	0.44 (0.39; 0.50)	0.20 (0.18; 0.23)
70 – <80 years	0.17 (0.15; 0.19)	0.26 (0.22; 0.29)	0.11 (0.09; 0.12)
80 – <90 years	0.08 (0.07; 0.10)	0.16 (0.13; 0.20)	0.05 (0.04; 0.06)
≥90 years	0.05 (0.03; 0.08)	0.13 (0.07; 0.24)	0.02 (0.01; 0.04)
Marital status (ref. married)			
single	1.16 (1.12; 1.20)	1.09 (1.04; 1.14)	1.29 (1.22; 1.36)
divorced	1.16 (1.09; 1.24)	1.27 (1.14; 1.42)	1.36 (1.24; 1.48)
widowed	2.14 (2.04; 2.24)	1.99 (1.85; 2.14)	2.28 (2.12; 2.44)
School education (ref. 10 years)			
<10 years	1.27 (1.24; 1.31)	1.27 (1.21; 1.32)	1.32 (1.26; 1.38)
>10 years	0.54 (0.52; 0.56)	0.54 (0.51; 0.57)	0.53 (0.50; 0.56)
Income (ref. <1000 DM)			
1000 DM – <2500 DM	1.21 (1.17; 1.26)	1.04 (0.98; 1.10)	1.21 (1.16; 1.26)
2500 DM – <4000 DM	1.07 (1.02; 1.11)	0.87 (0.81; 0.93)	1.18 (1.10; 1.26)
≥4000 DM	0.86 (0.81; 0.91)	0.69 (0.64; 0.75)	1.12 (1.00; 1.26)
Residency in East Germany (ref. West Germany)	1.02 (0.99; 1.05	1.05 (1.01; 1.10)	0.97 (0.93; 1.02)
**Size of community (ref. <20,000 inhabitants)**			
**20,000 – 500,000 inhabitants**	**1.17 (1.14; 1.20)**	**1.11 (1.07; 1.15)**	**1.25 (1.20; 1.30)**
**>500,000 inhabitants**	**1.56 (1.51; 1.62)**	**1.41 (1.34; 1.48)**	**1.76 (1.67; 1.86)**

**Logistic regression**, data are odds ratios (95% confidence intervals). All variables included as main factors.

100 DM = 51.13 Euro
